# Non-invasive low-cost deep tissue blood flow measurement with integrated Diffuse Speckle Contrast Spectroscopy

**DOI:** 10.3389/fnrgo.2023.1288922

**Published:** 2024-01-08

**Authors:** Arindam Biswas, Penaz Parveen Sultana Mohammad, Sadhu Moka, Arash Takshi, Ashwin B. Parthasarathy

**Affiliations:** Department of Electrical Engineering, University of South Florida, Tampa, FL, United States

**Keywords:** blood flow, diffuse optics, wearable sensing, spectroscopy, near infrared spectroscopy (NIRS), diffuse correlation spectroscopy (DCS)

## Abstract

Diffuse Correlation Spectroscopy (DCS) is a widely used non-invasive measurement technique to quantitatively measure deep tissue blood flow. Conventional implementations of DCS use expensive single photon counters as detecting elements and optical probes with bulky fiber optic cables. In recent years, newer approaches to blood flow measurement such as Diffuse Speckle Contrast Analysis (DSCA) and Speckle Contrast Optical Spectroscopy (SCOS), have adapted speckle contrast analysis methods to simplify deep tissue blood flow measurements using cameras and single photon counting avalanche detector arrays as detectors. Here, we introduce and demonstrate integrated Diffuse Speckle Contrast Spectroscopy (iDSCS), a novel optical sensor setup which leverages diffuse speckle contrast analysis for probe-level quantitative measurement of tissue blood flow. iDSCS uses a standard photodiode configured in photovoltaic mode to integrate photon intensity fluctuations over multiple integration durations using a custom electronic circuit, as opposed to the high frequency sampling of photon counts with DCS. We show that the iDSCS device is sensitive to deep-tissue blood flow measurements with experiments on a human forearm and compare the sensitivity and dynamic range of the device to a conventional DCS instrument. The iDSCS device features a low-cost, low-power, small form factor instrument design that will enable wireless probe-level measurements of deep tissue blood flow.

## 1 Introduction

Quantitative measurement of blood flow provides an index of blood perfusion, oxygenation, and nutrient supply to tissue areas, which is useful for monitoring tissue health (Durduran, 2004; Buckley et al., 2009; Yu, 2012a) and tracking disease development/healing (Baker et al., 2019). Over the last two decades, Diffuse Correlation Spectroscopy (DCS) (Boas and Yodh, 1997; Durduran and Yodh, 2014) has emerged as a popular non-invasive optical method to measure deep-tissue blood flow. DCS has been extensively validated (Carp et al., 2010; Buckley et al., 2012) and has been used in several applications such as imaging perfusion in breast cancers (Zhou et al., 2007; Yu, 2012b; Choe et al., 2014), monitoring muscle-tissue perfusion (Yu et al., 2005, 2007) and monitoring cerebral blood flow (Buckley et al., 2009, 2014; Carp et al., 2010; Durduran and Yodh, 2014) among others. DCS estimates blood flow from temporal fluctuations of diffuse photon intensities detected from the tissue surface, typically using single mode fibers and single photon counting modules. While the DCS system is overall robust and easy to use, single photon counting detectors are expensive and bulky, making conventional DCS instruments ill-suited for use as a low-cost or wearable device.

One simpler alternative is to use cameras as detecting elements, such as those typically employed in related photon correlation techniques such as Laser Speckle Contrast Imaging (LSCI) (Dunn, 2012). LSCI images the speckle pattern reflected from the tissue surface and analyzes spatial/temporal fluctuations therein to estimate blood flow. While LSCI instrumentation is simple to implement, its conventional implementations have limited depth sensitivity, restricted to single dynamic scattering measurement geometries. Recently, techniques such as Diffuse Speckle Contrast Analysis (DSCA) (Bi et al., 2013; Liu et al., 2017) and Speckle Contrast Optical Spectroscopy (SCOS) (Valdes et al., 2014; Dragojević et al., 2015) have extended LSCI to diffuse, deep-tissue blood flow measurements. While DSCA and SCOS utilize detection systems that are simpler and smaller than traditional DCS, these techniques still rely on Single Photon Counting Avalanche Photodiode Arrays (SPAD) or CCD/CMOS cameras that are still quite large for probe level measurements. In this contribution we extend these approaches to probe-level wearable sensing of deep tissue blood flow with a single unbiased, generic photo diode with a novel approach called integrated Diffuse Speckle Contrast Spectroscopy (iDSCS) (Biswas and Parthasarathy, 2020a,b). Use of a single photodiode permits a low noise, easy to operate device that requires a fraction of the power/bandwidth requirements of a camera. We demonstrate use of iDSCS on tissue simulating phantoms and compare its operation with conventional DCS systems in *in-vivo* experiments.

## 2 Materials and methods

### 2.1 Theory

We briefly review Diffuse Speckle Contrast Analysis (DSCA) (Bi et al., 2013; Valdes et al., 2014; Liu et al., 2017), which forms the theoretical basis for quantitative blood flow measurement with iDSCS. We note that DSCA is an extension of LSCI (Fercher and Briers, 1981; Bandyopadhyay et al., 2005; Boas and Dunn, 2010; Dunn, 2012) and Multi-Exposure Speckle Imaging (MESI) (Parthasarathy et al., 2008, 2010) applied to deep-tissue measurements. The primary difference between DCS and speckle contrast-based flow measurement methods is that former quantifies the temporal fluctuations in detected intensity as an autocorrelation function, while the latter computes the temporal variance of integrated photon intensities [*I*_*t*_(*T*)]. The two approaches are related via the speckle visibility expression.


(1) $$v\left(T,F\right)={\left(\frac{\sigma \left(T\right)}{\langle I_{t}\left(T\right)\rangle }\right)}^{2}=\frac{2\beta }{T}\int_{0}^{T}\left(1-\frac{\tau }{T}\right){\left[g_{1}\left(\rho ,F,\tau \right)\right]}^{2}d\tau$$

Here, *v*(*T, F*) is the normalized variance of the photon intensities (also referred to as speckle variance) measured over exposure/integration duration *T*, σ_*t*_ (*T*) and 〈*I*_*t*_〉 are the standard deviation and mean of *n* temporal samples of integrated intensity (typically *n* = 30), β is an instrumentation constant depending on speckle/detector size, coherence length and polarization of the light source (Bandyopadhyay et al., 2005), ρ is the source-detector separation, and τ is the correlation lag. *g*_1_(ρ, *F*, τ) is the normalized electric field autocorrelation function which for diffuse deep-tissue blood flow measurement is readily apparent from DCS literature (Boas and Yodh, 1997; Durduran and Yodh, 2014). Finally, an index of blood flow (*F*) is estimated by fitting Equation (1) to analytical solutions (Liu et al., 2017).

### 2.2 Instrumentation

**Figure 1:**
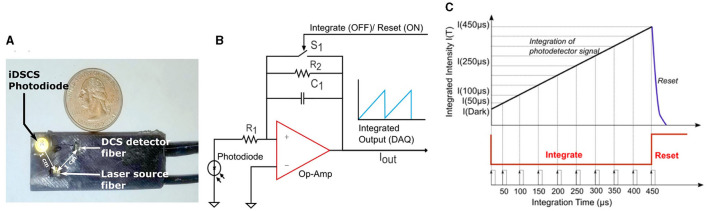
**(A)** Image depicting a representative iDSCS probe. It consists of a wavelength stabilized laser source and two detectors-iDSCS detector and a single mode fiber for conventional DCS measurements. The source detector separation for both detectors is 10 mm on this representative probe. **(B)** Circuit schematic of iDSCS detector. A generic photodiode is configured in photovoltaic mode. The current generated by incident photons is amplified by a programmable switched op-amp integrator circuit. The operation of the integrator is modulated by a FET switch on the feedback loop (S1), which controls the integration reset cycle (Typically *t*_*int*_ = 500 μs, *t*_*reset*_ = 100 μs). **(C)** Sampling and Integration cycle, for single shot measurement of speckle intensity fluctuations at multiple integration durations. The maximum integration time is 500 μs. Integration and data sampling is realized by a National Instruments data acquisition device controlled using custom software.

Figure 1 shows the instrumentation of an integrated Diffuse Speckle Contrast Spectroscopy (iDSCS) detector. The representative probe shown in Figure 1A is placed on the tissue surface. The probe features a fiber-coupled wavelength stabilized long coherence length laser source (Toptica Photonics, iBeam Smart, 785 nm, 120 mW), an iDSCS photodiode with associated circuitry, and a traditional DCS detection fiber. The probe was manufactured from silicone rubber based on a 3D printed template/mold (Baker et al., 2015). The source-detector separation for both iDSCS and traditional DCS systems was 1 cm for this representative probe; data from other source detector separations are also shown in this paper. Per traditional DCS, single mode detection fibers were coupled to single photon counting modules (SPCM-AQ4C, Excelitas) for computation of DCS intensity autocorrelation functions with a software correlator (Wang et al., 2016).

Figures 1B, C shows a schematic outline of the iDSCS sensor, instrument control and data acquisition. The iDSCS sensor uses a generic photodiode (ODD-1W, Optodiode, USA) that features an active detection area of ~1 *mm*^2^. The diode is operated in photovoltaic mode, i.e., it is unbiased. Normally, this reduces the photon-electron conversion rate and diminishes any amplification that may be achieved in the semiconductor. However, operating the photodiode in an unbiased condition also reduces the dark current in the sensor. The loss in photodiode amplification is compensated in the subsequent gain from the integrator amplifier circuit. The output of the photodiode, i.e., the current generated by the detected photons, is connected to a switched op-amp for integration. Here, we use a general-purpose precision switched integrator amplifier (IVC102U, Texas Instrument, USA) that features a capacitor bank and a MOSFET switch in feedback loop. The output of the amplifier can be modeled as: $\begin{array}{c} V_{out}=-\frac{1}{C_{int}}\int I_{IN}\left(t\right)dt \end{array}$, where, *C*_*int*_ = 100 *pF*, and *I*_*IN*_(*t*) is the current from the photodiode. When the MOSFET switch (S1) is open, the amplifier functions as an integrator, and the capacitor accumulates charge. When the MOSFET switch (S1) is closed by an external control signal, the integration operation is stopped and the capacitor discharges. Thus, the circuit functions as programmable integrator, like a camera. Note that the iDSCS circuit can be implemented with any operational amplifier with a capacitor and switch in its feedback loop.

The instrument control and data acquisition scheme shown in Figure 1C is realized using a multifunction data acquisition board (USB-6341, National Instruments, Austin, TX) and custom software (LabVIEW, National Instruments, Austin, TX). Two counter/timers on the data acquisition board were configured to generate the necessary timing signals to drive the acquisition process. The first timer was used to operate the integration/reset cycle (shown in red in Figure 1C). The integration time, *t*_*int*_, was set to be 500 μ*s* and the reset time, *t*_*reset*_ was set to 100 μ*s*. This pulse train was used to trigger the second timer and produce a periodic sampling clock, which was used to record the integrated intensities at different exposure/integration times. The sampling clock had a time period of 50 μ*s*, which resulted single-shot measurement of 10 exposures per cycle. A 500 μ*s* integration and 100 μ*s* reset cycle, resulted in an effective measurement frame rate of 1.6 *KHz*. Note that detection of additional exposures can be readily accomplished by reducing the time period of the sampling clock, without sacrificing data acquisition rates. Per data processing, *n* = 30 multi-exposure integrated intensity frames were processed to compute the speckle visibility curve [i.e., normalized speckle variance as a function of exposure duration, *v*(*T*)]. The speckle visibility data was then post-processed by averaging over 100 frames for an effective measurement rate of ~16 *Hz*. The resulting speckle visibility measurement was fit to an analytical solution to Equation 1, to derive a blood flow index. The number of intensity frames used to compute the speckle variance can influence the estimation of variance and is an important design consideration. Increasing the number of frames will improve variance estimates at the expense of overall measurement rate. As a compromise, our design uses 30 intensity frames (for temporal contrast). However, it should be noted that conventional speckle imaging studies suggest ~50 as an optimum number for spatial contrast (Duncan et al., 2008; Boas and Dunn, 2010).

### 2.3 Dynamic compensation of noise and speckle averaging

Since the iDSCS sensor utilizes an unbiased photodiode, it inherently features the lowest possible dark noise for Si detectors. However, the measurement is susceptible to other environmental and electrical noises such as Johnson noise, shot noise, readout noises etc. We implemented a real-time noise correction procedure to account for the different circuit noises. Note that the first sample acquired per cycle corresponds to a “zero” integration time measurement, and thus serves as an overall measure of circuit noises. This integrator output measured at zero integration time was subtracted from *I*_*IN*_(*T*) to provide a real-time noise corrected integrated intensity data for every cycle.

**Figure 2:**
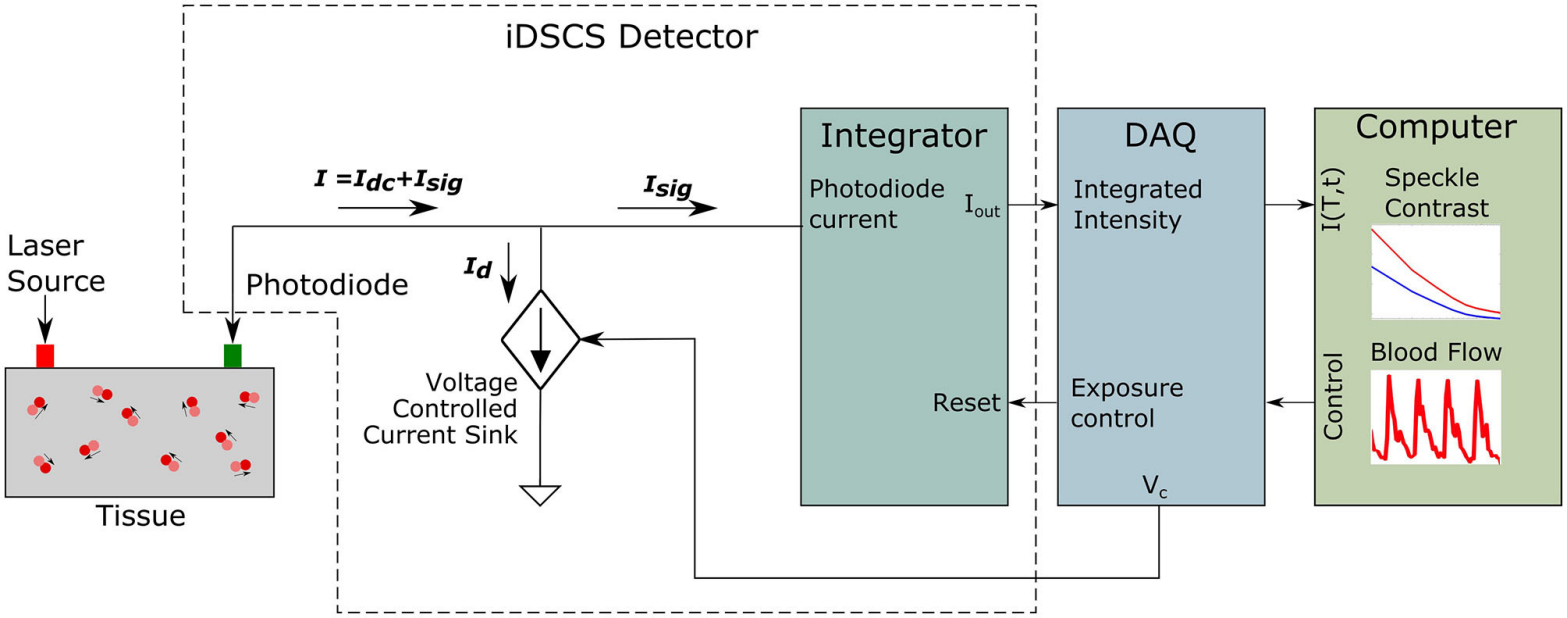
Schematic of the iDSCS detector with dynamic correction for speckle averaging. The total current from the photodiode is the addition of both current generated by speckle fluctuation *I*_*sig*_ and speckle averaging current *I*_*dc*_. The dc component is being subtracted by a programmable current drain *I*_*d*_ to improve measurement sensitivity.

A second consideration is speckle averaging at the detector. Conventional DCS instruments measure light using single mode fibers to detect light from a single speckle. In iDSCS, diffuse light from the sample is directly coupled to the photodiode. Since the photosensitive area of the photodiode is much larger than the speckle size, the iDSCS detector records a spatial average of multiple speckles, which reduces the resulting speckle contrast (Bandyopadhyay et al., 2005). If the current generated by the fluctuating photon intensities is *I*_*t*_ (*T*), we can model it to constitute a dc component contributed by the speckle averaging, *I*_*dc*_ (*T*), and an ac component, *I*_*sig*_ (*T*). When the dc current contribution is significantly higher that the ac component, i.e., *I*_*dc*_ (*T*)≫*I*_*sig*_ (*T*), the dynamic speckle contrast reduces, resulting in reduced dynamic range of blood flow measurement. Figure 2 outlines an approach for dynamic correction of speckle averaging at the iDSCS detector. To reduce the speckle averaging current *I*_*dc*_ (*T*), a constant current supply of magnitude *I*_*d*_ is configured as a current sink/drain to negate it. An op-amp driven constant current source is connected to the same node as the photodiode. The input terminal of the op-amp is connected to the analog output terminal of a data acquisition system (National Instruments NI-DAQ 6341). The output voltage of the NI-DAQ analog output terminal is converted to the drain current *I*_*d*_ by a series pull-down resistance. It is important to use a low-noise circuit to configure the drain current to ensure that fluctuations in the drain current do not add noise to the measurement. During experiment, *I*_*d*_ is controlled by software (LabVIEW); as *I*_*d*_ approaches *I*_*dc*_, the spackle variance increases. Increased speckle variance allows smaller changes in the speckle visibility curve to be measured which leads to better dynamic range.

## 3 Experiments and results

### 3.1 iDSCS measurements of blood flow in human arm

We first demonstrate the ability of the iDSCS technique to measure deep tissue blood flow in humans. All *in-vivo* experiments were performed according to human subject protocols approved by the Institutional Review Board at the University of South Florida. Briefly, we acquired iDSCS speckle variance data by attaching the optical probe (similar to Figure 1A) to the forearm of a healthy volunteer. The probe was secured to the arm using tape, and measurements were made at two separate source detector separation 1 cm and 2.5 cm. The overall time period of acquisition was set to be *t*_*acquisition*_ = 600 μ*s* where the integration time *t*_*int*_ was set to be 500 μ*s* and the reset time *t*_*reset*_ was set to be 100 μ*s*. Ten multi-exposure intensity/contrast measurements were collected for exposure times ranging from 0 μ*s* to 450 μ*s*.

**Figure 3:**
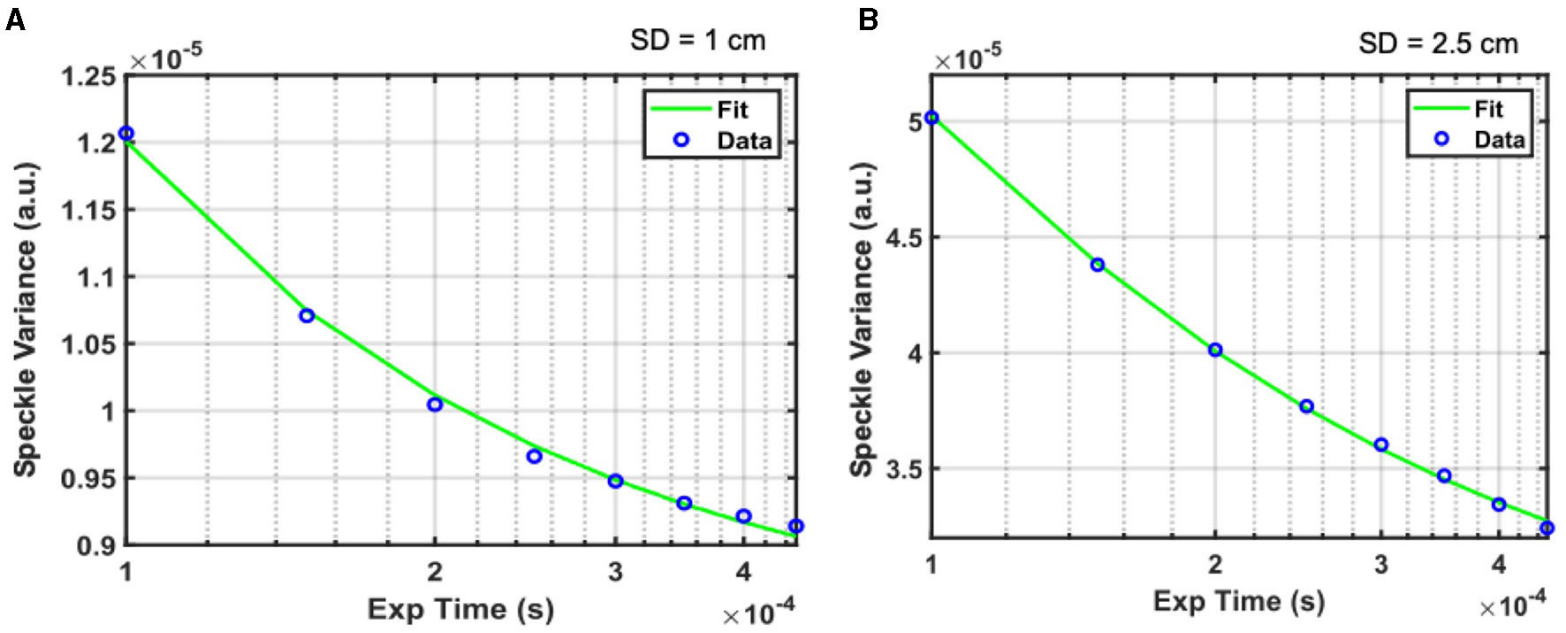
Representative speckle variance curves acquired under baseline conditions with the iDSCS probe for 1 cm **(A)** and 2.5 cm **(B)** SD separation. Blue circles are measured speckle variance data, green line is the fir of the data to the diffuse speckle contrast model to estimate blood flow.

Representative speckle variance curves measured during baseline are shown in Figure 3 for source-detector separations of 1 cm (Figure 3A) and 2.5 cm (Figure 3B). The speckle variance at 1 cm is lower compared to the speckle variance at 2.5 cm for the same drain current introduced i.e., 0.05 μ*A*. This is mainly due to inadequate compensation of speckle averaging at 1 cm source-detector separation, which would see a significantly higher measured intensity. At shorter 1 cm SD the light reflection from the tissue contributed to higher intensity and overall greater (spatial) speckle averaging than the longer SD separation thus compromising the speckle variance. The green lines indicate fits of the speckle variance data to the diffuse speckle model (Liu et al., 2017). A 100-frame moving average was performed on the data before fitting.

### 3.2 Validation of dynamic compensation for speckle averaging

**Figure 4:**
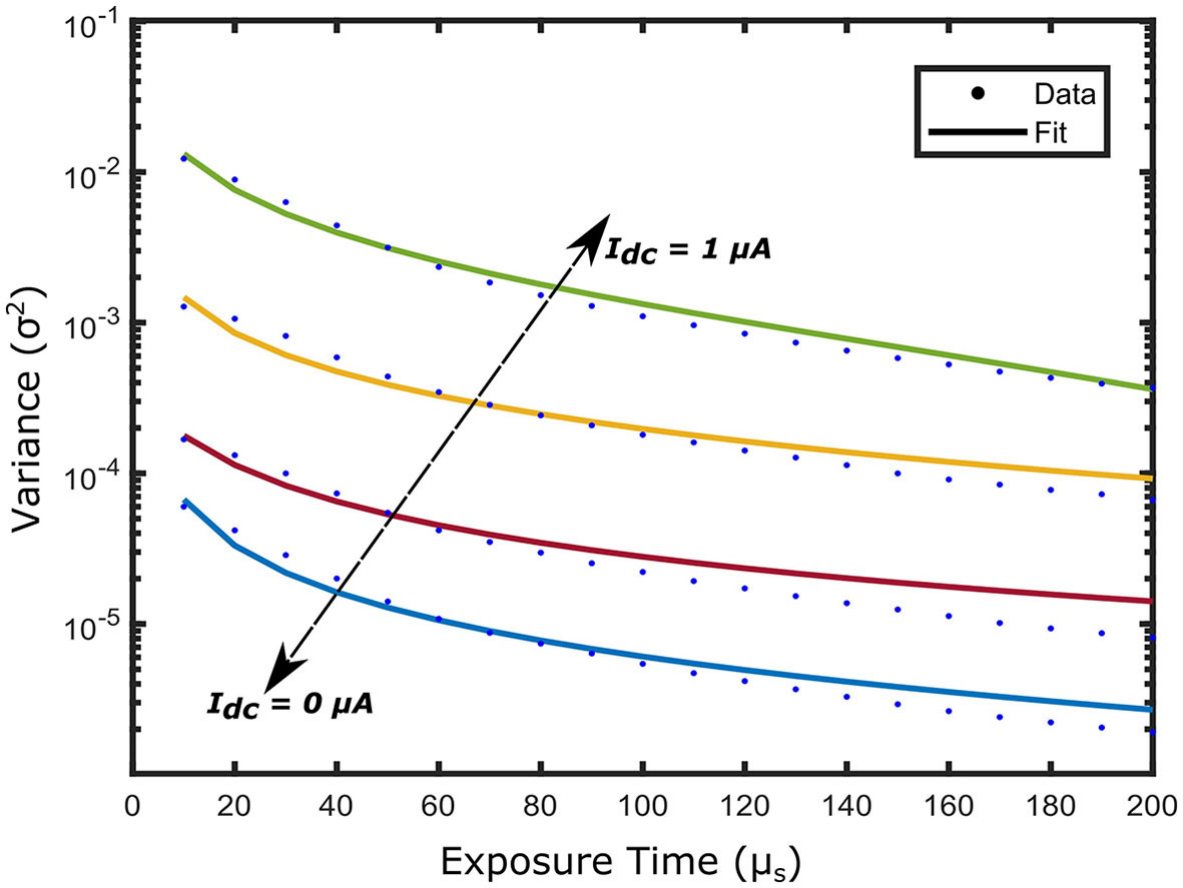
Speckle variance curves acquired from a solid phantom experiment by varying the drain current *I*_*d*_. Different current sink *I*_*d*_ is introduced to the terminal of the photodiode ranging from 0 μ*A* to 1 μ*A*. As the *I*_*d*_ increases the dynamic range of the speckle variance curve improves reaching a peak at *I*_*d*_ = 1 μ*A*, denoting a total subtraction of the speckle averaging current *I*_*dc*_.

An important aspect of the experimental demonstration of the iDSCS probe is the dynamic compensation technique for speckle averaging effects. We performed a liquid tissue phantom experiment to validate this approach. We measured diffuse speckle visibility curves from a liquid tissue phantom ($\mu_{a}=0.1\;cm^{-1}$, $\mu_{s}=10\;cm^{-1}$) with the iDSCS optical probe with a source detector separation of ρ = 1 cm. Multiple integrated speckle intensity curves were recorded as a function of exposure time *T* from 10μ*s* to 200μ*s*. A total of 100 such curves were used to calculate the speckle variance. Speckle variance was acquired as a function of exposure time for drain currents ranging from 0 μ*A* to 1 μ*A*; Figure 4 outlines speckle variance curves measured with drain currents (*I*_*d*_) of 0 μ*A* (blue), 0.5 μ*A* (red), 0.9 μ*A* (yellow) and 1 μ*A* (green). The average speckle contrast is lowest when *I*_*d*_ = 0 μ*A* and highest when *I*_*d*_ = 1 μ*A*; speckle variance measured at *I*_*d*_ = 1 μ*A* is three orders of magnitude greater than that measured without drain current subtraction. These results clearly show the success of the dynamic contrast enhancement strategy implemented here.

### 3.3 Sensitivity of iDSCS to measure blood flow changes, compared to DCS

**Figure 5:**
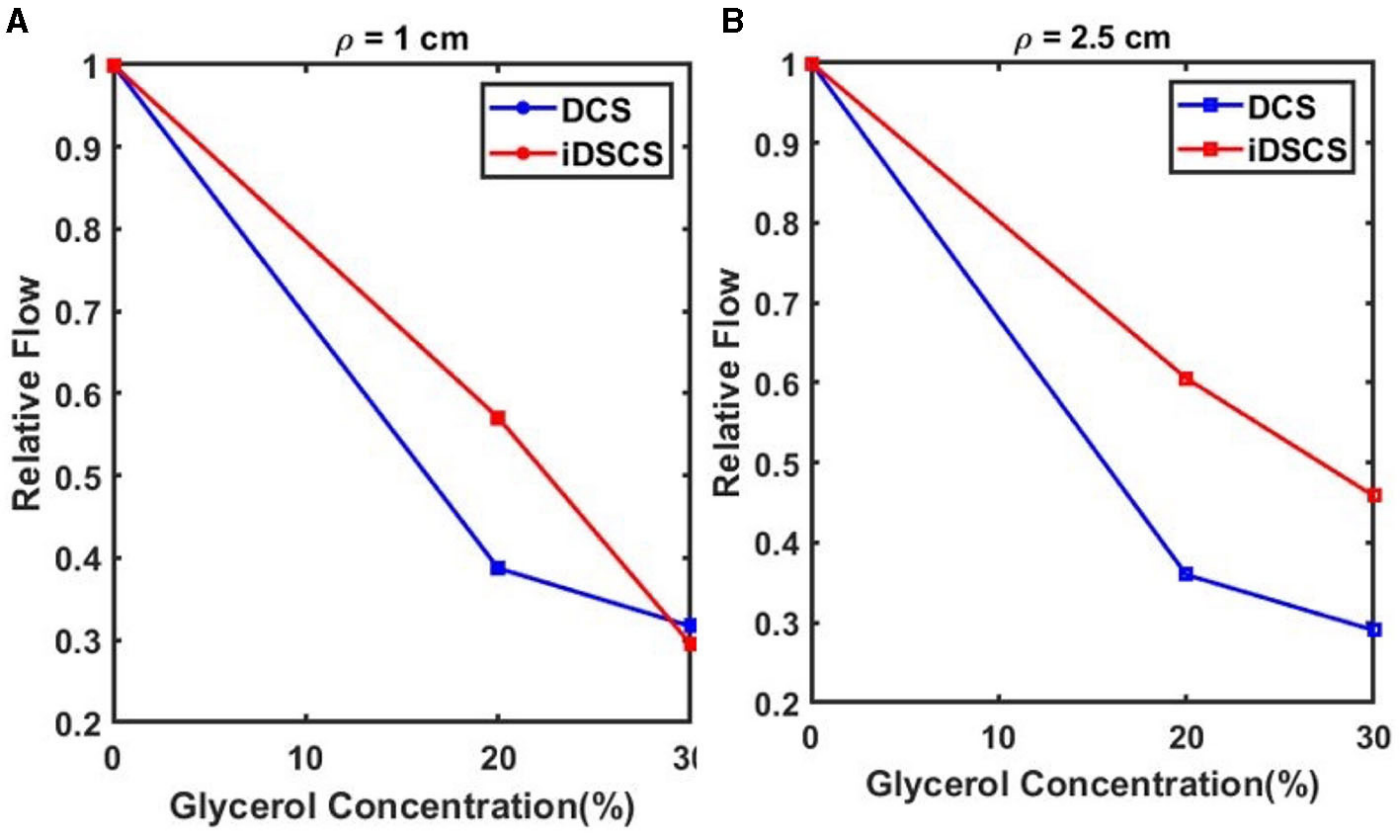
Sensitivity of flow changes measured with iDSCS (red lines) and DCS (blue lines) for 1 cm **(A)** and 2.5 cm **(B)** source detector separation. Here, the blood flow index measured on the phantom with 0% glycerol was used as a reference. Both iDSCS and DCS track a decrease in flow index with increase in viscosity. DCS measures a larger flow decrease compared to iDSCS.

Next, we performed a tissue phantom experiment to compare the sensitivity of DCS and iDSCS instruments to measure blood flow changes. To simulate flow changes in a controlled manner we prepared a tissue simulating liquid phantom with suspended scattering molecules at different viscosities, i.e., a liquid phantom consisting of water, intralipid (20% emulsion, Sigma-Aldrich, MO) and glycerol (Sigma-Aldrich Co. LLC. U.S., cod. 49770, ≥99.5%). The intralipid acts as the scattering agent whereas the water to glycerol ratio changes the viscosity. We prepared solutions with three different water to glycerol concentration ranging from 0% to 30% (v/v) following the same process described in an earlier study (Cortese et al., 2018). Similar to Figure 1A, the iDSCS probe used in this experiment comprised of two source fibers maintaining 1 cm and 2.5 cm separations from a fiber coupled DCS detector (conventional DCS) and an embedded iDSCS sensor. DCS and iDSCS data were taken for 1 min simultaneously for each solution with different glycerol concentration. The measured speckle visibility curves (iDSCS) and autocorrelation functions (DCS) were averaged and fit to their respective models to estimate the blood flow index for the different phantoms/modalities. Figure 5A shows the relative flow changes for an SD separation of 1 cm measured with iDSCS (red) and DCS (blue), whereas Figure 5B shows the relative flow measured at SD separation of 2.5 cm. Here, the flow index measured at 0% glycerol was used as a reference point. In both figures, from left to right as the glycerol concentration increases, the viscosity increases, decreasing the speed of suspended scatterers (i.e., intralipid molecules). At both 1 cm and 2.5 cm, both iDSCS and DCS show decreases in blood flow. However, the iDSCS measures a slightly smaller decrease in flow compared to DCS. This indicates that the iDSCS system may have a lower sensitivity to flow changes compared to DCS.

**Figure 6:**
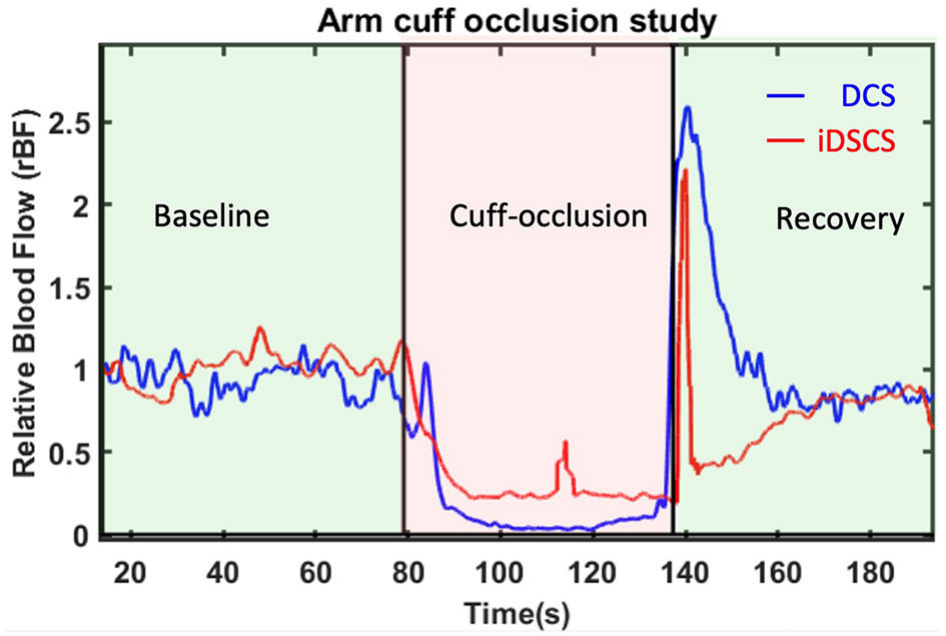
Relative blood flow changes due to arm-cuff occlusion measured with DCS and iDSCS. Source-detector separation of both measurements was 2.5 cm. Both DCS and iDSCS track the almost 100% reduction in blood flow due to arm-cuff occlusion. iDSCS flow changes are less sensitive than DCS for large flow changes.

We extended the preceding sensitivity experiment to *in-vivo* tissues. In an experiment similar to that described earlier, DCS and iDSCS were used to measure deep tissue blood flow changes from a human arm. To induce transient blood flow reduction in the forearm, a blood pressure cuff was wrapped around the bicep. The cuff-occlusion protocol consisted of ~1-min baseline, followed by ~1-min cuff ischemia where the blood pressure cuff was inflated to 200 mmHg, and finally a 1-min post occlusion recovery. Experiments were performed at a source detector separation of 2.5 cm. The DCS acquisition frequency was set to 20 Hz and an average of 20 frames was taken to demonstrate the relative change in blood flow. Three channels of DCS data were averaged to increase the SNR. For the iDSCS measurement the integration time was set to *t*_*int*_ = 300 μ*s* with reset time *t*_*reset*_ = 10 μ*s* resulting in an effective measurement rate of 3.225 *kHz*. During processing a series of 30 frames were used to calculate the variance curves thus making the effective frame rate to 107 *Hz*. Before fitting into the speckle variability model, a window average of 100 frames was performed. Measured blood flow indices were averaged with a 20-frame moving average window. For both measurements the blood flow indices were normalized with the mean of flow in the baseline period to compute the relative blood flow (rBF). The results of the experiment are shown in Figure 6. Both the measurement techniques are in good agreement with each other and show reduction in blood flow during occlusion. However, there is around 10–15% discrepancy in relative blood flow during the occlusion period between the two methods. We note that this difference could arise from the positional difference and lack of sensitivity due to speckle averaging and other measurement non-linearity.

## 4 Discussion

In this paper, we have introduced a new optical sensor for measurement of deep-tissue blood flow using a generic photodiode and custom electronic circuits. The new method, integrated Diffuse Speckle Contrast Spectroscopy, was demonstrated to be sensitive to blood flow changes in the human arm and was compared for range and sensitivity against conventional Diffuse Correlation Spectroscopy (DCS). Over the past 2–3 decades, DCS has grown to be a standard method for deep tissue blood flow measurements, but it's wide-spread commercial and clinical adoption, à la functional Near Infrared Spectroscopy (fNIRS) has been stunted in part due to the somewhat strict hardware requirements. Recently, we showed that the high coherence length requirement for DCS laser light sources (e.g., wavelength stabilized diode lasers) can be relaxed, and that fast blood flow measurements can be performed without loss of signal-to-noise using generic single mode diode lasers placed directly onto the probe, i.e., illuminating the tissue without a fiber optic cable (Biswas et al., 2021). Here, we have significantly relaxed the hardware requirements for the detector and show that deep-tissue blood flow measurement is possible with a low-cost photodiode. These instrumental improvements enable deep tissue blood flow measurements using low-cost laser diodes and photodiodes; these simple components can be arranged/configured into low-weight wearable optdodes that are similar to the current state-of-the-art in functional Near Infrared Spectroscopy. Indeed, these technical advances bring forth the feasibility of whole-head imaging of cerebral blood flow, and for wearable ambulatory monitors of cerebral blood flow in humans.

The iDSCS photodiode utilizes theory and simple instrumentation prevalent in Laser Speckle Contrast Imaging. This idea has been previously explored with technologies such as Diffuse Speckle Contrast Analysis (DSCA) (Liu et al., 2017) and Speckle Contrast Optical Spectroscopy (SCOS) (Valdes et al., 2014). Our work is thematically similar but novel in instrument design. Instead of using a SPAD array or a camera, iDSCS detects light with a single photodiode with a custom integration circuit. This approach offers several advantages. First, due to its small footprint, the iDSCS sensor can be readily incorporated within an optical probe for fiber-less detection of blood flow. Second, the iDSCS sensor permits single-shot measurement of speckle variance at multiple exposure times, which is an advantage over similar camera-based devices. Third, the unbiased operation of the photodiode and dark current measurements provides real-time noise correction and customization. Finally, the sensor's low power/bandwidth requirements permit embedded measurements with microcontrollers. It can conceivably be powered by a battery for wireless operation (Stiner, 2021).

Nevertheless, we note a few important limitations of the current iteration of the iDSCS device. Most significantly, the sensitivity and dynamic range of iDSCS to changes in blood flow is lower than conventional DCS, primarily due to the speckle averaging effect. The detection area of iDSCS photodiode is an order of magnitude larger than detection area of a single pixel of cameras or single mode detection in conventional DCS instruments. As a result, the detrimental effect of speckle averaging at the detector is amplified for the iDSCS technique. We have presented a dynamic compensation approach to counteract speckle averaging, but this is dependent on the average intensity measured by the iDSCS photodiode. More research is needed to optimize this process and improve the sensitivity and dynamic range of the iDSCS device.

Finally, we discuss noise considerations for blood flow measurements with iDSCS. Like camera-based speckle contrast blood flow measurements, the variance measured with iDSCS can be affected/biased by different noise sources including shot noise, dark noise and readout noise (Valdes et al., 2014; Zilpelwar et al., 2022). The larger detection areas used in the iDSCS device allows more light to be collected by the photodiode compared to conventional DCS or cameras. Thus, the operating currents of the iDSCS device is often well above the noise floor of the photodiode; as such there is currently no shot noise correction such as those suggested elsewhere (Valdes et al., 2014). Anecdotally, we have noticed that shot noise correction does not significantly alter our blood flow estimates. Nevertheless, fully characterizing and modeling the effects that changes in drain current (which reduces the overall photodiode current) impart on the shot noise, and implementing noise corrections, may help improve the sensitivity of the iDSCS device. Correction for dark-current is inherent in the operation of the iDSCS device. Furthermore, since the iDSCS photodiode is unbiased, the dark-current is minimum. Unlike camera-based devices, there is no readout operation for the iDSCS device. However, failure to discharge the feedback capacitor during reset (see Figure 1B) may result in a readout-like noise. To elaborate, the iDSCS device works by integrating the measured photocurrent with a programmable integrator with a feedback capacitor. At the end of an integration cycle, the capacitor is discharged by short-circuiting the feedback circuit. If the capacitor is not fully discharged, any residual charge will add to the measured intensity in the next integration cycle. Since the residual charge can randomly change from cycle to cycle, this could lead to an added variance/bias to the measured speckle variance. This readout-like noise variance can be minimized by ensuring that the reset time is large enough to fully discharge the feedback capacitor. Therefore, in contrast to camera-based instruments, noise variances in the iDSCS device can be reduced by hardware control. However, any noise performance benefits of the iDSCS device needs to be examined in a systematic manner.

## Data availability statement

The raw data supporting the conclusions of this article will be made available by the authors, without undue reservation.

## Ethics statement

The studies involving humans were approved by University of South Florida Institutional Review Board. The studies were conducted in accordance with the local legislation and institutional requirements. The participants provided their written informed consent to participate in this study.

## Author contributions

AB: Conceptualization, Data curation, Formal analysis, Investigation, Methodology, Software, Validation, Writing – original draft. PM: Investigation, Writing – review & editing. SM: Investigation, Writing – review & editing. AT: Conceptualization, Supervision, Writing – review & editing. AP: Conceptualization, Funding acquisition, Methodology, Project administration, Supervision, Validation, Writing – review & editing.
